# Cancer Chemoprevention by Phytochemicals: Nature’s Healing Touch

**DOI:** 10.3390/molecules22030395

**Published:** 2017-03-03

**Authors:** Haseeb Zubair, Shafquat Azim, Aamir Ahmad, Mohammad Aslam Khan, Girijesh Kumar Patel, Seema Singh, Ajay Pratap Singh

**Affiliations:** 1 Department of Oncologic Sciences, Mitchell Cancer Institute, University of South Alabama, Mobile, AL 36604, USA; HZubair@health.southalabama.edu (H.Z.); SAzim@health.southalabama.edu (S.A.); AAhmad@health.southalabama.edu (A.A.); MAKhan@health.southalabama.edu (M.A.K.); GPatel@health.southalabama.edu (G.K.P.); SeemaSingh@health.southalabama.edu (S.S.); 2 Department of Molecular Biology and Biochemistry, College of Medicine, University of South Alabama, Mobile, AL 36688, USA

**Keywords:** phytochemicals, cancer chemoprevention, tumor microenvironment, inflammation, pro-oxidant, nanotechnology

## Abstract

Phytochemicals are an important part of traditional medicine and have been investigated in detail for possible inclusion in modern medicine as well. These compounds often serve as the backbone for the synthesis of novel therapeutic agents. For many years, phytochemicals have demonstrated encouraging activity against various human cancer models in pre-clinical assays. Here, we discuss select phytochemicals—curcumin, epigallocatechin-3-gallate (EGCG), resveratrol, plumbagin and honokiol—in the context of their reported effects on the processes of inflammation and oxidative stress, which play a key role in tumorigenesis. We also discuss the emerging evidence on modulation of tumor microenvironment by these phytochemicals which can possibly define their cancer-specific action. Finally, we provide recent updates on how low bioavailability, a major concern with phytochemicals, is being circumvented and the general efficacy being improved, by synthesis of novel chemical analogs and nanoformulations.

## 1. Introduction

Cancer is currently the second leading cause of death worldwide and a major health problem throughout the world. It is estimated that in 2017, the United States alone will have 1,688,780 new cancer diagnoses and 600,920 cancer-related deaths [1]. While significant progress has been made to improve diagnosis and surveillance, this has not helped much to improve the overall cancer survival rates. This had led to a surge in molecular targeted therapies to develop better clinical outcomes for cancer patients [2]. Unfortunately, these strategies have also not provided substantial improvement due to the development of resistance against therapies [3], reviving an interest in the prospects of phytochemicals, natural anticancer agents from plants, due to the multitude of effects of these agents on diverse molecular signaling pathways, with no or minimal toxicity in normal cells [4].

It is believed that the number of new cancer cases can be reduced and many cancer-related deaths can be prevented. The studies focused on ‘cancer prevention’ are a step in this direction. The overall aims of cancer prevention are broad. The *primary aim* is to completely prevent or at least delay the onset of cancer through maintenance of healthy lifestyle, avoidance of exposure to toxicants/carcinogens, and dietary consumption of chemopreventive agents and drugs. The *secondary aim* depends on the early detection of cancer in the precancerous or early stage tumors, thereby helping in the better management and treatment of these tumors, and the *tertiary aim* of cancer prevention involves reducing the risk of metastases, development of secondary tumors and recurrence, using preventive agents. Natural products (from botanicals, herbs, etc.) as well as minerals and vitamins have been demonstrated to affect all three areas of cancer prevention [5,6]. Among natural products, phytochemicals represent the class of compounds that have been extensively studied for their biological effects.

Consumption of fruits and herbal medicines in the diet is a convenient and effective method of administering phytochemicals in a cost-effective manner [7,8]. Overall, at least 20% of all cancers can be prevented by consumption of diets rich in vegetables and fruits (>400 g/day) [9]. According to an analysis, of all the 175 small molecules approved for cancer therapy from 1940s to the year 2014, 85 (49%) were natural products or directly derived therefrom [4]. Phytochemicals continue to enter clinical trials or provide leads for the synthesis of semi-synthetic medicinal agents. However, the laboratory success of phytochemicals has not been reproduced in the clinic. This is partly due to the inherent differences between in vitro laboratory experiments and the human physiological conditions. As with other drugs, improvements in formulation of compounds with potential anticancer properties are being made for better therapeutic outcomes. In this article, we briefly discuss the mechanism of action of phytochemicals and, instead of touching upon the classical activities such as induction of apoptosis/cell cycle arrest, we discuss the effect of phytochemicals on reactive oxygen species (ROS) production and the tumor microenvironment (TME), along with updates on current studies to improve the efficacy of these compounds.

## 2. Preventive and Therapeutic Mechanisms of Phytochemicals

**Figure 1:**
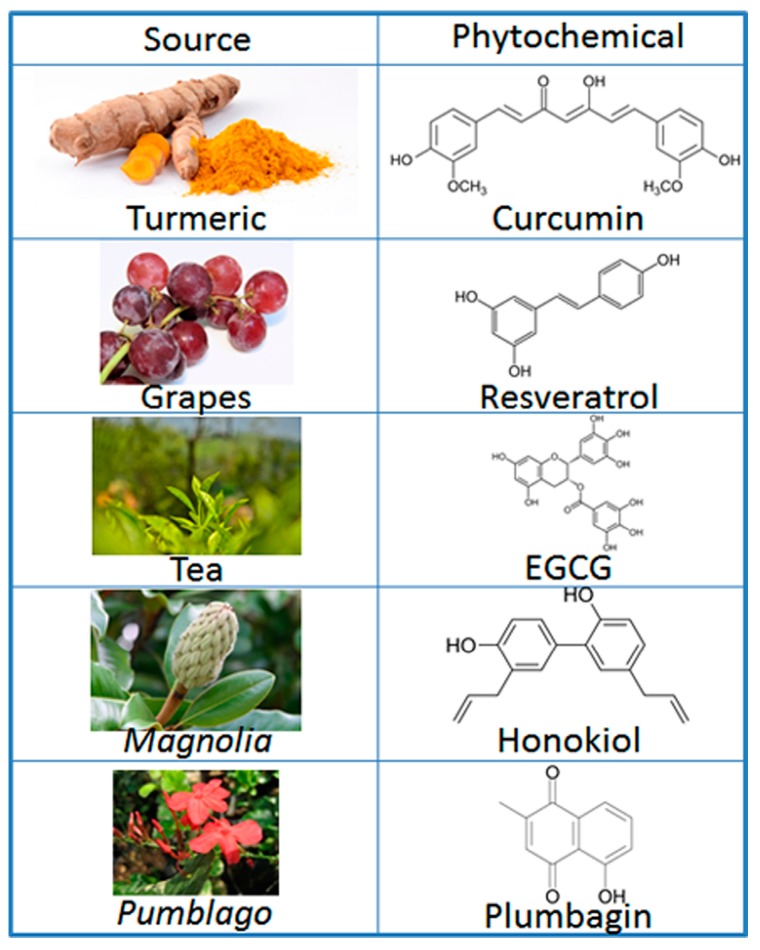
Phytochemicals and their sources. EGCG: Epigallocatechin gallate.

The proposed mechanisms by which vegetables and fruits affect human cancers are multiple and complex. Various stages of carcinogenesis may be inhibited, and various in vitro or in vivo systems are used to model these inhibitory effects in preclinical studies. Therefore, characterizing the active chemical components of these plant products and accumulation of compelling in vitro and animal study data prior to clinical studies is necessary. Phytochemicals, due to their dietary origin, are presumed safer and are better tolerated with relatively low toxicity. For the ease of discussion and to keep the discussion focused, we have chosen five representative phytochemicals, *viz*. curcumin, epigallocatechin-3-gallate (EGCG), resveratrol, plumbagin and honokiol. Chemical structures of these phytochemicals are provided in Figure 1.

### 2.1. Modulation of Oxidative Stress

Healthy cells maintain an intricate balance of redox homeostasis wherein the levels of ROS and reactive nitrogen species (RNS) are fine*-*tuned by the antioxidant defense system [10]. ROS/RNS are by-products of normal cell metabolism with physiological roles at moderate and low concentrations [10]. Oxidative stress is induced as a result of imbalance in the redox status of the cells, and has been suggested to be responsible for the pathophysiology of several diseases, including cancer. In cancer, oxidative stress by physical and chemical agents, inflammation and infection can give rise to direct DNA damage inducing tumorigenesis [11]. Oxidative stress also alters protein conformation and function, thus affecting regular functions of affected proteins. Phytochemicals are generally known for their antioxidant activity and have been demonstrated to counteract the damaging effects of oxidation in vitro by a direct quenching of ROS. In addition, phytochemicals also upregulate the expression of genes that detoxify reactive species, metabolize toxic compounds, and maintain cellular homeostasis.

The ability of phytochemicals to inhibit chemically-induced carcinogenesis in mice models has been a cornerstone for their chemopreventive property. A delay in tumor promotion has been shown, upon concurrent topical application of 12-*O*-tetradecanoylphorbol-13-acetate (TPA) and phenolic compounds such as caffeic acid, chlorogenic acid, curcumin and ferulic acid [12], or resveratrol and ursolic acid [13]. TPA is a commonly used phorbol diester, a known tumor promoter, employed to over-activate the PKC signaling, and is a potent generator of superoxide anions [14]. Thus, the ability of phytochemicals to inhibit/delay the progression of TPA-induced carcinogenesis is indicative of their antioxidant effects. Plumbagin [15] and honokiol [16] have also been reported to inhibit TPA-induced effects. In addition, the promotion and progression of 4-nitroquinoline-1-oxide (4-NQO)-induced tongue carcinogenesis in rats was inhibited by these polyphenols when administered in the diet [17]. 4-NQO is a carcinogenic agent that introduces DNA damage through the production of ROS [18]. Curcumin, the most bioactive constituent of turmeric and an integral part of the Indian diet, inhibits diethylnitrosamine (DEN)-induced hepatocarcinogenesis in mice at a concentration of 0.2% in the diet [19]. DEN specifically induces carcinogenesis of the gastrointestinal tract, especially liver, through the downregulation of ROS-detoxifying enzymes [20]. Green tea and black tea constituents have also demonstrated potential inhibition of 7,12-dimethylbenz[a]anthracene (DMBA)-treated UVB-induced skin carcinogenesis [21]. Administration of decaffeinated green or black tea to mice has been reported to significantly reduce 4-(methylnitrosamine)-1-(3-pyridyl)-1 butanone-induced tumor formation in mice [22]. 

### 2.2. Inhibition of Inflammation

The persistent inflammation and inflammatory mechanisms have been implicated as the basis of several diseases, such as chronic conditions of old age [23]. Studies have also provided significant evidence to demonstrate a positive relation between inflammation and cancer [24,25]. Chronic inflammatory states are triggered by multiple factors such as microbial infections, obesity, autoimmune diseases, etc. [26]. Underlying infections or inflammatory responses have been linked to nearly 15%–20% of cancer-associated deaths [27]. A number of phytochemicals have been suggested to interfere with inflammation-related pathways, partially explaining their anti-cancer potential [28] (Table 1). Curcumin is a well-known anti-inflammatory agent [29]. Its effect on inflammation is mediated by multiple mechanisms, such as transforming growth factor beta1 (TGF-β1) up-regulation and down-regulation of inducible nitric oxide synthase (iNOS) and cyclooxygenase-2 (COX2) [30], as well as by modulation of toll-like receptors (TLR)/interleukin-1 receptor (IL-1R) pathway [31]. Resveratrol, another known anti-cancer agent [32,33], also works as an anti-inflammatory agent [34]. It inhibits the production of pro-inflammatory cytokines [interleukin (IL)-6/8 and tumor necrosis factor-alpha (TNF-α)] and pro-inflammatory miR-155, while also inducing the anti-inflammatory cytokines and miR-663 [35]. Resveratrol has also been shown to interfere with pro-inflammatory events triggered by IL1-β [36].

A number of signaling pathways play a critical role in inflammation, and often connect inflammation with cancer onset and progression. Signaling through nuclear factor kappa-light-chain-enhancer of activated B cells (NF-κB) is one well studied pathway that links inflammation with cancer [49]. It also happens to be the pathway that has been investigated in detail, with respect to the anticancer effects of phytochemicals [45,50,51,52]. Almost all phytochemicals have been shown to affect NF-κB signaling, including curcumin, resveratrol, EGCG, plumbagin and honokiol [42,44,45,46,47,48,53]. The Kelch-like erythroid cell-derived protein with Cap’n’collar homology-associated protein (Keap1)/NF-E2 p45-related factor 2 (Nrf2) pathway is another pathway that regulates inflammation-related gene expression [54]. Nrf2 coordinates antioxidant response to several stimuli, thereby preventing oxidative damage and onset of inflammation-responsive diseases, including cancer. Keap1, under normal conditions, keeps Nrf2 sequestered in the cytoplasm thus preventing Nrf2 from discharging its antioxidant functions. It is therefore proposed that targeting of Keap1 by phytochemicals can favorably induce the antioxidant activity of Nrf2. Indeed, several phytochemicals are well known to target Keap1/Nrf2 pathway [30,37,39,40,41,55].

### 2.3. Prooxidant Activity

While antioxidant activity of phytochemicals has traditionally been at the forefront of investigations into their putative anticancer action, interestingly, many of these agents also exhibit prooxidant action, particularly in the presence of transition metal ions, especially copper [33,56,57]. There seems to be enough evidence to support phytochemicals-mediated production of ROS, a prooxidant action that is responsible for their ability to induce apoptosis in cancer cells [58,59]. Several phytochemicals that are antioxidants at some concentrations become prooxidants at other concentrations. The relevance of copper in the prooxidant action of phytochemicals stems from the observation that preneoplastic and neoplastic cells have elevated copper levels, compared to normal cells [60]. Such cancer cells with endogenously elevated copper levels are much more sensitive to electron transfer, and generation of ROS, presumably through redox recycling of copper ions [32,56]. Therefore, the prooxidant action of phytochemicals, in the presence of redox active transition ions, represents an important pathway through which transformed cells are selectively targeted by phytochemicals while normal cells survive. Such prooxidant action has been demonstrated for many phytochemicals, including curcumin [61,62], EGCG [59], plumbagin [63] and resveratrol [32]. Copper is not the only metal ion with associated prooxidant activity. Iron [64] and zinc ions [65] have also been reported to generate ROS leading to prooxidant activity. Interestingly, the anticancer activity of vitamin C [66,67] has also been reported to involve prooxidant production of ROS. Further, a role of vitamin C in Fenton reaction type production of ROS, involving redox recycling on iron ions, is well known [68]. An investigation into prooxidant activity of known antioxidants and vitamins reported prooxidant potential of vitamins A and C in synergy with iron and copper ions, with combination of vitamin C and copper being the most effective [69]. Based on these evidences, it is conceivable that the prooxidant mechanism of induction of apoptosis better explains the anticancer effects of phytochemicals, through generation of ROS close to the DNA. It also helps explain how anticancer agents with diverse chemical structures function similarly, and exhibit preferential cytotoxicity against cancer cells. 

### 2.4. Modulation of Tumor Metabolism

Metabolism in tumor cells is intricately connected with ROS production [70]. Cancer cells extensively alter their metabolic activity to meet the growing needs associated with survival and growth [71]. The two most important metabolites supporting tumor growth are glucose and glutamine [72]. Breakdown of glucose supports energy demand and the generation of biosynthetic metabolites for the growth of cancer cells. In addition, glutamine, the most abundant amino acid in human plasma, not only contributes to the energy pool, but also provides nitrogen for the biosynthesis of nitrogen-containing compounds. Thus, targeting the altered tumor cell metabolism can yield therapeutic outcomes. 

A number of studies have now identified the regulation of glucose and glutamine metabolism by phytochemicals through different mechanisms [73,74]. Phytochemicals have been observed to directly inhibit the basal transport of glucose in cancer cells to improve their response to chemotherapy [75]. Curcumin, in addition to glucose uptake, has been shown to alter glutathione as well as lipid metabolism in association with docetaxel [76]. The ability of curcumin to interfere with glucose transport can be explained by its direct binding [77] and inhibition of glucose transporter 1 (GLUT1) [78]. Similar to curcumin, resveratrol [79] and plumbagin [80] can also down-regulate GLUT1. As a proof that glucose metabolism can be effectively targeted in cancer cells, particularly in the cancer cells with metastatic mesenchymal phenotype, glucose-coated magnetic nanoparticles were observed to be preferentially taken up by mesenchymal cells, as opposed to epithelial cells [81]. Blocking of GLUT1 affected the uptake of nanoparticles, suggesting an important role of glucose shell on the nanoparticles. In addition to curcumin [82,83] and resveratrol [83,84], EGCG [85,86], plumbagin [87] and honokiol [88] have also been reported to alter glucose metabolism leading to anti-cancer effects. Given the relevance of glutamine metabolism in cancer cells, phytochemicals have also been tested for their ability to modulate glutamine metabolism. It was reported that resveratrol-induced cell death in castration-resistant C4-2 prostate cancer cells depends on glutamine metabolism [89]. Further, curcumin’s cytotoxicity against colorectal cancer stem cells was suggested to involve blocking of glutamine’s entry into the cells though coupling of curcumin to the CD44 receptors [90]. Thus, there is evidence in literature to support an effect of phytochemicals on glucose and glutamine metabolism as a mechanism for their anti-tumor properties.

The predominance of aerobic glycolysis in cancer cells, the ‘Warburg effect [91]’ has generated a lot of attention in last several decades. Interestingly, phytochemicals have been shown to reverse this phenomenon. Curcumin could reverse inflammatory TNF-α mediated Warburg effect in breast cancer cells [92]. Resveratrol was recently shown to partially reverse Warburg effect resulting in increased cell death through increased oxygen consumption, hyperpolarization of mitochondrial membrane and ROS generation [93]. While the contribution of ‘Warburg effect’ to tumorigenesis has been challenged in recent years [94], particularly with the realization that a number of tumor cells have functional mitochondria, the overall role of altered metabolism in tumor cells still remains an attractive target for therapy [95]. With multi-targeted effects against different metabolic pathways, phytochemicals are prime candidates for further testing in clinical settings.

## 3. Modulation of Tumor Microenvironment

The tumor microenvironment (TME) refers to the immediate vicinity of tumor cells that is populated by many different types of cells/factors: immune cells, fibroblasts, cytokines, blood vessels, etc. The dynamic interactions between several components within the TME are now considered drivers of cancer progression [96]. The bi-directional talk between tumor cells and the surroundings in the TME facilitates their proliferation, invasion and metastasis, in addition to bestowing upon them the ability to evade therapeutic insults. In view of the important role that the TME plays in tumor progression, it is critical for any putative therapy to be able to modulate the TME favorably, countering the many advantages that the TME confers to the tumor cells. There are reports documenting the effects of phytochemicals on the TME as basis of their anti-cancer activity [97,98].

In pancreatic cancer the TME is particularly marked by severe hypoxia, which, in turn, triggers the activation of hedgehog (Hh) signaling [99,100]. The cross-talk between tumor cells and the surrounding stroma in the hypoxic TME has a profound dependence on Hh signaling [100] making Hh signaling an attractive target for therapy. A recent report has documented the inhibitory action of curcumin against Hh signaling in hypoxic pancreatic cancer cells Panc-1 TME [101]. In addition to its action against Hh signaling, curcumin was also demonstrated to reverse hypoxia-induced epithelial to mesenchymal transition (EMT). Resveratrol is also a potent inhibitor of Hh signaling, as revealed by its action in hypoxic TME of pancreatic cancer cells BxPC-3 and Panc-1, through a mechanism that involved suppression of ROS [102]. Similar to these effects of curcumin and resveratrol, our own investigations have revealed an effect of honokiol against Hh signaling in pancreatic cancer TME [103]. In addition to Hh signaling, we found a potent effect of honokiol against C-X-C chemokine receptor type 4 (CXCR4), another important factor that mediates tumor-stromal cross-talk. Further, EGCG can decrease hypoxia in non-small cell lung cancer (NSCLC) A549 cells through a rebalance of angiopoietins, resulting in sensitization of these cells to cisplatin [104].

In addition to induction of EMT, there is also evidence for enrichment of cancer stem cells (CSCs) in the TME, which can be effectively targeted by curcumin [105]. Not just in a pancreatic cancer model, CSCs are also enriched in the TME of colorectal cancer as well, where curcumin is able to interfere with the cross-talk between CSCs and stromal fibroblasts, resulting in reversal of EMT and the associated metastasis [106]. In the colorectal TME, the therapeutic potential of curcumin is underlined by its ability to potentiate 5-FU activity [106,107]. The same research group has reported a very similar action of resveratrol as well [108] which also involves potentiation of 5-FU activity in a 3D-alginate microenvironment, through reversal of EMT and inhibition of NF-κB signaling.

There is also evidence for immunomodulatory potential of phytochemicals within the TME. For example, curcumin, when delivered as polyethylene glycol (PEG) conjugate along with Trp2 peptide vaccine, resulted in reduced IL-6 levels and down-regulation of immunosuppressive factors (regulatory T cells, myeloid-derived suppressor cells) in a melanoma-bearing mouse model [109]. Resveratrol, however, in a renal cell carcinoma model, could only reduce regulatory T cells, but had no effect on myeloid-derived suppressor cells [110]. Further, COP9 signalosome 5 (CSN5) stabilized programmed cell death-ligand-1 (PD-L1) plays important role in TNF-α-induced cancer immunosuppression in TME, and curcumin can inhibit CSN5, leading to sensitization of cancer cells to immunotherapy [111]. 

Plumbagin seems to be a promising phytochemical to target bone metastasis of breast cancer [112] because of its ability to target tumor-bone microenvironment [113]. It specifically disrupts association of receptor activator of nuclear factor of κB (RANK) with TNF receptor-associated factor 6 (TRAF6), thus abrogating mitogen-activated protein kinases (MAPK) and NF-κB signaling [113]. IL-6 levels can be reduced by EGCG in breast cancer TME, as part of regulation of tumor-associated macrophages (TAMs) [114], and IL-18 can be inhibited by resveratrol in melanoma TME, leading to reduced metastasis [115]. Also, plumbagin has been demonstrated to inhibit the activity of c-MYB [116], an important modulator of tumor-stromal cross-talk and pancreatic cancer signaling [117]. In addition, EGCG has been shown to inhibit prostate cancer-associated myofibroblast differentiation [118]. Thus, there seems to be ample evidence in support of an action of phytochemicals against various components of the TME (Table 2).

## 4. Challenges for Phytochemicals in Cancer Therapy and Emerging Alternatives

Despite many promises and the demonstrated success in in vitro and pre-clinical studies, there has been little to no progress in the transition of phytochemicals to the clinic as the first line therapy. The limitations of in vitro testing models are well known. A direct exposure of cancer cell lines during in vitro testing causes an acute presentation of phytochemicals, inducing significant anticancer and antiproliferative action at concentrations usually not achieved under normal physiological conditions even upon consumption of the pure compound extract. While these in vitro studies provide significant insights into the cellular signaling mechanisms, they do not provide information on the effect of test agent on the organism as a whole. However, the obvious practical and ethical limitations of involving human studies without substantial laboratory evidence still makes it mandatory to rely on such in vitro models as the first step. Thus, there is a pressing need for in vitro and/or preclinical models that can mimic systemic exposure to phytochemicals, with resulting metabolomic and pharmacokinetic changes. 

The first and foremost challenge is the problem of bioavailability [119,120,121]. Since a majority of these phytochemicals are part of normal human diet, they are efficiently metabolized and cleared by body. They do not persist in physiological systems and the therapeutic effects are usually short-lived [119]. The other aspect of using phytochemicals in cancer therapy is the lack of target specificity. While this is viewed by some as a limitation, it is increasingly being realized that such lack of specificity, and the multi-targeted effects of phytochemicals, the ‘pleiotropic’ effects, underline the very essence of these anticancer agents [51,122], particularly in view of the knowledge that when challenged with targeted therapies, tumor cells often activate alternate pathways which results in failure of targeted therapy. Under such circumstances, a multi-targeted therapeutic agent is likely to be a comparatively more effective because of its ability to check the activation of alternate survival pathways.

While the issue of bioavailability cannot be resolved just by increasing the administered dose or the frequency of administration, there are certain alternate ways which are being pursued to circumvent this problem. These include: (a) chemical syntheses of novel analogs of phytochemicals to increase the efficacy and bioavailability; (b) novel formulations to selectively and more effectively deliver the phytochemicals to their intended target organs; and (c) formulation of novel delivery systems that modulate the pharmacokinetics of the anticancer agent. In this section, we will highlight some recent advancements in these fields, particularly related to the phytochemicals discussed so far, to provide an overview of the broader research field.

### 4.1. Synthesis of Chemical Analogs

Curcumin is an exemplary phytochemical that has shown lots of potential in pre-clinical studies, only to fail in clinical settings. It also remains one of the most extensively modified phytochemicals, with so many reported analogs that it will be beyond the scope of this article to comment on every single curcumin analog that has been tested and reported for its enhanced anticancer activity. Just to put this into perspective, some curcumin analogs (Table 3) reported within the past two years include C-150 (inhibits NF-κB in glioblastoma cells [123]), Da0324 (inhibits NF-κB in gastric cancer cells [124]), 2,2’-fluoromonocarbonyl analog (modulates ROS in lung cancer cells [125]), A17 (induces ER stress in lung cancer cells [126]), MC37 (induces cell cycle arrest in colorectal cancer cells [127]), HO-3867 (STAT3 inhibitor in pancreatic cancer cells [128]), BDMC-A (inhibits NF-κB in breast cancer cells [129]), GO-Y078 (inhibits invasion of endothelial cells [130]), DM-1 (induces apoptosis in melanoma cells [131]), FLLL12 (induces apoptosis in lung cancer cells [132]), BHBA (activates Nrf2 in lung cancer models [133]) and L49H37 (induces apoptosis in pancreatic stellate cells [134]). Other than these, there are a few other curcumin analogs that have been investigated in comparatively more detail. These include difluorinated curcumin (CDF, inhibits MMP2 in lung cancer cells [135], restores PTEN in colorectal cancer cells [136] and inhibits cancer stem cells in pancreatic and colorectal cancer models [137,138]), WZ35 (modulates ROS in gastric cancer [139,140] and prostate cancer cells [141]) and EF24 (inhibits src phosphorylation in hepatocellular carcinoma cells [142], suppresses EMT through miR-33b in melanoma cells [143], induces ROS-dependent apoptosis in colorectal cancer cells [144], suppresses NF-κB in cholangiocarcinoma [145] and induces apoptosis in pancreatic cancer cells [146]).

Resveratrol has also generated considerable interest because of its anti-cancer potential, and consequently, a number of groups have synthesized analogs of resveratrol with an aim to enhance its efficacy [147,148,149,150,151,152,153] (Table 3). The DMU-212 analog of resveratrol has not just been reported to exhibit enhanced anti-tumor effects, but, interestingly, its mechanism of action has been reported to be distinct from the parent compound, resveratrol [154]. DMU-212 has also been suggested to suppress pro-inflammatory factors, especially NF-κB [155], a mechanism which happens to be similar to resveratrol. The interest in DMU-212 has led to synthesis of its own analogs that have been tested against a panel of 60 human cancer cell lines with mixed results [156]. HS-1793 is another synthesized analog of resveratrol with multiple reported properties ([157,158,159,160,161,162]).

In contrast to curcumin and resveratrol, only a handful of studies have reported synthesis of EGCG analogs. The d-ring analog of EGCG was one of the first to be made, and it was observed to target VEGF in breast cancer cells [163]. Such VEGF-targeting activity of another EGCG analog, a methylated one, has also been reported [164]. Other analogs of EGCG include the fluoro-substituted analogs that have shown promise in inhibiting proteasomal activity of leukemia [165] and breast cancer cells [166,167]. There is evidence for synthesis of novel plumbagin analog [168] but it has not yet been tested in pre-clinical cancer models. For the phytochemical honokiol, a dichloroacetate ester has been synthesized which inhibits androgen receptor signaling in prostate cancer cells [169] and re-sensitizes vemurafenib-resistant melanomas [170].

### 4.2. Novel Formulations

Nanotechnology has expanded the horizon of anti-cancer therapy in general [171], and the activity of several phytochemicals in particular [172]. Due to the overwhelming interest in curcumin in pre-clinical studies, a number of reports are available on the nanoformulations of curcumin as well as its analogs, all with the premises of recapitulating the pre-clinical success of curcumin in clinical settings [173,174,175]. Again, similar to the sub-section above, we will list here reports from only last two years, to keep the discussion meaningful.

Poly(d,l-lactic acid)-glycerol (PDLLA)-G-based curcumin nanoparticles have been reported to be as effective as free curcumin in in vitro assays, and their in vivo clearance was comparatively lower [176]. Such delayed clearance of curcumin nanoparticles can result in enhanced in vivo activity, as confirmed in a cervical cancer model [177]. Curcumin-loaded monomethoxy polyethylene glycol (mPEG)- poly(ε-caprolactone) (PCL) micelles showed increased plasma retention [178] while curcumin’s oligosaccharide of hyaluronan conjugate nanoparticle has been shown to be more stable and less toxic, relative to curcumin [179]. Similarly, curcumin-cyclodextrin/cellulose nanocrystal complexes kill colorectal and prostate cancer cells at IC_50_ values lower than curcumin [180]. Poly(lactic-co-glycolic acid) (PLGA)-curcumin particles have been demonstrated to deliver active curcumin directly to the cells’ cytosolic compartment, resulting in enhanced therapeutic activity [181]. This is similar to amphiphilic polyaspartamide polyelectrolytes that increase cellular uptake of curcumin [182]. Targeted delivery to prostate cancer tissue has been reported via lipid-polymer hybrid nanoparticles that encapsulate curcumin as well as docetaxel [183]. Conjugating curcumin on the hydrophilic terminals of pluronic F68 chains through *cis-*aconitic anhydride linkers is proposed to improve delivery of curcumin [184] while curcumin micelles have been shown to overcome multidrug resistance [185].

Not only the parent compound curcumin, there has been interest in nanoformulation of curcumin analogs as well. Nanomicelles of curcumin analog curcumin difluorinated (CDF) with amphiphilic styrene-maleic acid copolymer (SMA) increased the solubility by 5%–15% and exhibited enhanced antitumor effect [186] while liposome encapsulation of CDF sensitized the cisplatin-resistant head and neck cancer stem cells [187]. The hyaluronic acid-SMA-CDF micelles were reported to specifically target pancreatic cancer stem cells [188], similar to hyaluronic acid-PAMAM dendrimer formulation of CDF [189]. Another curcumin analog, EF24, has also been nanoencapsulated in pegylated liposomes resulting in enhanced anticancer effects against pancreatic cancer cells in vitro and in a pancreatic xenograft model [190]. Pegylated curcumin nanoparticles have also shown promise in real-time monitoring of drug release [191]. Another concept of embedding phytochemicals in the biopolymer PCL to continuously deliver the small molecule for extending periods of time has also been demonstrated for the delivery of curcuminoids [192]. The PCL-implant leads to the release of the agent in a two-step process. An initial burst releases the drug present on the surface into circulation. This initial burst-release can be controlled by coating an empty polymer; followed by the slow-steady release of the agent present in the matrix. Moreover, the site of implantation is hypothesized to increase drug accumulation at the site and a controlled release [192]. 

There are reports on nanoformulations of resveratrol as well. Resveratrol’s PCL and PLGA-PEG-COOH nanoparticles have been shown to improve release of resveratrol in hypoxia-relevant acidic TME, with efficient take up by the prostate cancer cells [193]. One of the objectives of nanoformulations is to reduce effective toxicity and this was reported in case of lipid nanoparticles of resveratrol when tested in lung cancer cells exposed to cigarette smoke condensate [194]. Another objective is to increase solubility and this has been demonstrated by nanocomplexation of resveratrol with soy protein isolate [195]. The gold nanoparticles of resveratrol have been shown to be biologically active with activity against multiple signaling cascades [196].

Efforts have also been made to formulate EGCG nanoparticles. In an early report on the topic [197], EGCG was conjugated with FITC, and the complex could enter the cytoplasm as well as the nucleus. In another report, gelatinized EGCG was observed to retain its biological activity against breast cancer cells, and was as potent as EGCG [198]. Similarly, PLA-PEG-EGCG nanoparticles also retained their activity, and even reported a 10-fold dose advantage in in vitro as well as in in vivo assays [199,200]. While free EGCG was completely degraded in 4 hours, the EGCG nanoparticles had a significantly longer half-life [201,202]. Co-encapsulation of EGCG with paclitaxel within a targeted PLGA-casein nanoparticle has been reported to re-sensitize paclitaxel-resistant breast cancer cells [203].

For the phytochemical plumbagin, silver nanoparticles were synthesized to overcome the lack of sensitivity and selectivity towards cancer cells, and such formulation reduced the effective concentration required to induce apoptosis to half of the free compound [204] in addition to enhancing the cellular uptake [205]. The reduced toxicity in normal cells, and better bioavailability of nanoformulated plumbagin has been reported by another independent group as well [206]. There has been some interest in nanoformulations of honokiol as well. The PCL-PEG-PCL-honokiol nanoparticles were shown to release honokiol over extended time [207]. The MPEG-PLA-encapsulation also had similar effect on honokiol release [208] and, moreover, this formulation also made honokiol injectable [209]. The stability of honokiol was remarkably improved by MPEG, to the extent that whereas it took 2 h to release 20% honokiol from the formulation in plasma, it took more than 200 h to release the same amount of honokiol in PBS [210].

Recently nanoformulations of bioactive lipids have been described. These lipids, such as the fatty acid docosahexaenoic acid, have been observed to demonstrate potent anticancer properties against a variety of cancer types such as breast cancer, ovarian cancer, glioblastomas, etc. [211,212,213,214]. The nanocapsules, comprising shells of bioactive lipids, are able to deliver drugs and other biologically sensitive molecules to specific cells or organs, thus enhancing the potency and cancer therapeutic potential [211,215]. This represents a promising area of novel nanoformulation where some progress has been made in recent years [216,217]. As a whole, the developments in the field of nanotechnology have raised the hopes of using phytochemicals as chemotherapeutic anticancer agents in near future.

## 5. Conclusions and Perspectives

**Figure 2:**
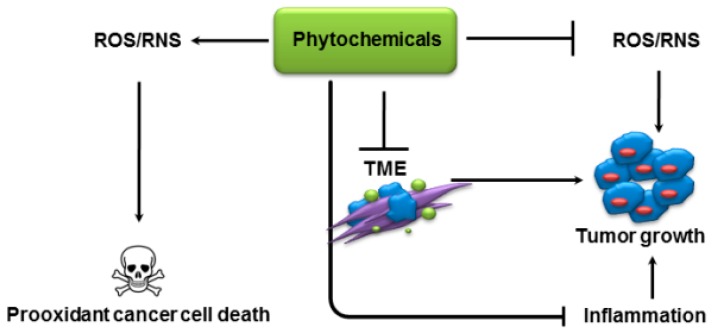
Role of phytochemicals in human malignancies. Phytochemicals potentially scavenge reactive oxygen species (ROS) or upregulate anti-oxidant signalling to combat ROS generation in cancer cells to inhibit growth. There are also context dependent evidences which advocate the prooxidant cell death inducing behaviour of phytochemicals. Phytochemicals have also been shown to inhibit inflammation via targeting NF-κB pathway. Tumor micro environment (TME) plays a vital role in many solid tumors pathogenesis, and phytochemicals have been shown to target both tumor and stromal compartments.

The action of phytochemicals against cancer cells, via inhibition of proliferation, invasion, angiogenesis and metastasis, is well documented. A number of derived analogs have also been tested in different pre-clinical models. New investigations into phytochemicals’ mechanism of action have suggested that many of the observed pre-clinical effects of phytochemicals can possibly be explained by their ability to regulate TME (Figure 2). The importance of TME in cancer progression is well recognized. However, the studies involving regulation of TME and its various components can get quite challenging, with unavailability of assay systems that can accurately mimic the complexities of TME. This is specially challenging for studies with phytochemicals, where often there is a need for quick and efficient screening of several novel phytochemicals or their synthetic analogs. As a step in this direction, a 3-dimensional microfluidic device has been reported, with a capability of assaying multiple compounds simultaneously for their anti-metastatic potential [218]. The co-culture of endothelial and cancer cells in this device, and their 3-dimensional morphology, better represents TME. Such advancements in experimental capabilities provide hope for possible use of phytochemicals in clinics in near future.

Nanotechnology has been used to enhance chemoprevention outcome and the resulting ‘nanochemoprevention’ [219] seems to be relevant to enhancing the efficacy of phytochemicals [201]. However, nanoparticles themselves are often toxic, and often not suitable for oral consumption. This has led to novel ways to prepare nanoparticles, like for example chitosan-based nanoparticles [220]. Further, the individual chemicals used in nanoformulations are not alike, and differ in their ability to reinforce the intended biological effect. For example, silver –based nanoparticles are superior than zinc and titanium-based nanoparticles in protection against UV-induced DNA damage [221]. Thus, the promises of nanotechnology rest on fine tuning and careful characterization of the underlying materials and methods. Interestingly, the concept of ‘green’ nanotechnology, using biodegradable and eco-friendly materials, is gaining ground [222,223]. The combination of green nanotechnology and the natural phytochemicals sounds promising. By producing phytochemicals, nature has provided a healing touch to the very problems it frequently presents, including diseases such as cancer. Persistent and innovative methods to improve the anti-cancer efficacy of phytochemicals should not halt, until the pre-clinical success of these agents is realized in clinics.

## Figures and Tables

**Table 1:** Inflammation-influencing signaling factors/pathways modulated by phytochemicals.

Signaling Factor/Pathway	Phytochemical	Reference
**COX2**	Curcumin	[30]
**IL-1β**	Resveratrol	[36]
**IL-6**	Resveratrol	[35]
**IL-8**	Resveratrol	[35]
**iNOS**	Curcumin	[30]
**TLR/IL-1R**	Curcumin	[31]
**Keap1/Nrf2**	Curcumin	[30]
	EGCG	[37,38]
	Honokiol	[39]
	Plumbagin	[40]
	Resveratrol	[41]
**NF-κB**	Curcumin	[42]
	EGCG	[43,44]
	Honokiol	[45,46]
	Plumbagin	[47]
	Resveratrol	[48]

**Table 2:** Tumor microenvironment components affected by phytochemicals.

TME Component	Phytochemical	Cancer Model	Reference
CSCs	Curcumin	Pancreatic	[105]
		Colorectal	[106]
Hh signaling	Curcumin	Pancreatic	[101]
	Honokiol	Pancreatic	[103]
	Resveratrol	Pancreatic	[102]
CXCR4	Honokiol	Pancreatic	[103]
IL-6	Curcumin	Melanoma	[109]
	Resveratrol	Renal	[110]
	EGCG	Breast	[114]
IL-18	Resveratrol	Melanoma	[115]
Microvasculature	EGCG	NSCLC	[104]
Myofibroblast Differentiation	EGCG	Prostate	[118]
RANK	Plumbagin	Breast	[113]
Regulatory T-cells	Curcumin	Melanoma	[109]
	Resveratrol	Renal	[110]

**Table 3:** Chemical analogues of phytochemicals and their reported effects.

Phytochemical	Analogue	Reported Activity	Reference
Curcumin	C-150	Inhibits NF-κB	[123]
	Da0324	Inhibits NF-κB	[124]
	2-2′-fluorine mono-carbonyl analog	Modulates ROS	[125]
	A17	Induces ER stress	[126]
	MC37	Induces cell cycle arrest	[127]
	HO-3867	Inhibits STAT3	[128]
	BDMC-A	Inhibits NF-κB	[129]
	GO-Y078	Inhibits invasion of endothelial cells	[130]
	DM-1	Induces apoptosis	[131]
	FLLL12	Induces apoptosis	[132]
	BHBA	Activates nrf2	[133]
	L49H37	Induces apoptosis in pancreatic stellate cells	[134]
	CDF	Multiple pathways affected	[135,136,137,138]
	WZ35	Modulates ROS	[139,140,141]
	EF24	Multiple pathways affected	[142,143,144,145,146]
EGCG	D-ring analog	Targets VEGF	[163]
	Methylated analog	Targets VEGF	[164]
	Fluoro-substituted	Inhibits proteasomal activity	[165,166]
Honokiol	Dichloroacetate ester	Inhibits AR, chemosensitizes	[169,170]
Plumbagin	Isoniazid analog	Multiple pathways affected	[168]
Resveratrol	DMU-212	Inhibits NF-κB	[155]
	HS-1793	Multiple pathways affected	[157,158,159,160,161,162]

## Abbreviations

The following abbreviations are used in this manuscript:

4-NQO	4-nitroquinoline-1-oxide
5-FU	5-FluoroUracil
CDF	Curcumin Difluorinated
COX2	CycloOxygenase2
CSC	Cancer Stem Cells
CSN5	COP9 Signalosome 5
CXCR4	C-X-C chemokine receptor type 4
DEN	DiEthylNitrosamine
DMBA	7,12-Dimethylbenz[a]anthracene
EGCG	EpiGalloCatechin Gallate
EMT	Epithelial-Mesenchymal Transition
FITC	Fluorescein IsoThioCyanate
Hh	Hedgehog
IL	InterLeukin
iNOS	inducible Nitric Oxide Synthase
Keap1	Kelch-like erythroid cell-derived protein with Cap’n’collar homology-associated protein
MAPK	Mitogen-Activated Protein Kinases
MMP2	Matrix MetalloProteinase2
MPEG	Mono methoxy Poly Ethylene Glycol
NF-κB	Nuclear Factor Kappa-light-chain-enhancer of activated B cells
Nrf2	NF-E2 p45-related factor 2
NSCLC	Non-Small Cell Lung Cancer
PCL	poly (ε-CaproLactone)
PD-L1	Programmed cell Death-Ligand-1
PDLLA-G	Poly(d,l-lactic acid)-glycerol
PEG	PolyEthylene Glycol
PKC	Protein Kinase C
PLA	Poly Lactic Acif
PLGA	Poly(Lactic-co-Glycolic Acid)
PTEN	Phosphatase and Tensin Homolog
RANK	Receptor Activator of Nuclear Factor of κB
RNS	Reactive Nitrogen Species
ROS	Reactive Oxygen Species
SMA	Styrene-Maleic Acid copolymer
TGF-β	Transforming Growth Factor beta1
TLR	Toll-Like Receptors
TME	Tumor MicroEnvironment
TNF-α	Tumor Necrosis Factor-Alpha
TPA	12-*O*-tetradecanoylphorbol-13-acetate
TRAF6	TNF Receptor Associated Factor 6
UV	UltraViolet
VEGF	Vascular Endothelial Growth Factor

## References

[1] Siegel R.L., Miller K.D., Jemal A. (2017). Cancer statistics, 2017. CA Cancer J. Clin..

[2] Okimoto R.A., Bivona T.G. (2014). Recent advances in personalized lung cancer medicine. Per Med.

[3] Krepler C., Xiao M., Sproesser K., Brafford P.A., Shannan B., Beqiri M., Liu Q., Xu W., Garman B., Nathanson K.L. (2016). Personalized preclinical trials in braf inhibitor-resistant patient-derived xenograft models identify second-line combination therapies. Clin. Cancer Res..

[4] Newman D.J., Cragg G.M. (2016). Natural products as sources of new drugs from 1981 to 2014. J. Nat. Prod..

[5] Janakiram N.B., Mohammed A., Madka V., Kumar G., Rao C.V. (2016). Prevention and treatment of cancers by immune modulating nutrients. Mol. Nutr. Food Res..

[6] Chih H.J., Lee A.H., Colville L., Binns C.W., Xu D. (2013). A review of dietary prevention of human papillomavirus-related infection of the cervix and cervical intraepithelial neoplasia. Nutr. Cancer.

[7] Terry P., Wolk A. (2001). Tea consumption and the risk of colorectal cancer in sweden. Nutr. Cancer.

[8] Van Duyn M.A., Pivonka E. (2000). Overview of the health benefits of fruit and vegetable consumption for the dietetics professional: Selected literature. J. Am. Diet. Assoc..

[9] Tseng M. (2009). Diet, cancer and public health nutrition. Public Health Nutr..

[10] Blokhina O., Fagerstedt K.V. (2010). Oxidative metabolism, ros and no under oxygen deprivation. Plant Physiol. Biochem..

[11] Reuter S., Gupta S.C., Chaturvedi M.M., Aggarwal B.B. (2010). Oxidative stress, inflammation, and cancer: How are they linked?. Free Radic. Biol. Med..

[12] Huang M.T., Smart R.C., Wong C.Q., Conney A.H. (1988). Inhibitory effect of curcumin, chlorogenic acid, caffeic acid, and ferulic acid on tumor promotion in mouse skin by 12-*o*-tetradecanoylphorbol-13-acetate. Cancer Res..

[13] Cho J., Rho O., Junco J., Carbajal S., Siegel D., Slaga T.J., DiGiovanni J. (2015). Effect of combined treatment with ursolic acid and resveratrol on skin tumor promotion by 12-*o*-tetradecanoylphorbol-13-acetate. Cancer Prev. Res. (Phila).

[14] Blumberg P.M. (1988). Protein kinase c as the receptor for the phorbol ester tumor promoters: Sixth rhoads memorial award lecture. Cancer Res..

[15] Shieh J.M., Chiang T.A., Chang W.T., Chao C.H., Lee Y.C., Huang G.Y., Shih Y.X., Shih Y.W. (2010). Plumbagin inhibits tpa-induced mmp-2 and u-pa expressions by reducing binding activities of nf-kappab and ap-1 via erk signaling pathway in a549 human lung cancer cells. Mol. Cell Biochem..

[16] Konoshima T., Kozuka M., Tokuda H., Nishino H., Iwashima A., Haruna M., Ito K., Tanabe M. (1991). Studies on inhibitors of skin tumor promotion, ix. Neolignans from magnolia officinalis. J. Nat. Prod..

[17] Yanaida Y., Kohno H., Yoshida K., Hirose Y., Yamada Y., Mori H., Tanaka T. (2002). Dietary silymarin suppresses 4-nitroquinoline 1-oxide-induced tongue carcinogenesis in male f344 rats. Carcinogenesis.

[18] Nunoshiba T., Demple B. (1993). Potent intracellular oxidative stress exerted by the carcinogen 4-nitroquinoline-n-oxide. Cancer Res..

[19] Chuang S.E., Kuo M.L., Hsu C.H., Chen C.R., Lin J.K., Lai G.M., Hsieh C.Y., Cheng A.L. (2000). Curcumin-containing diet inhibits diethylnitrosamine-induced murine hepatocarcinogenesis. Carcinogenesis.

[20] Ajiboye T.O., Komolafe Y.O., Bukoye Oloyede H.O., Yakubu M.T., Adeoye M.D., Abdulsalami I.O., Oladiji A.T., Akanji M.A. (2013). Diethylnitrosamine-induced redox imbalance in rat microsomes: Protective role of polyphenolic-rich extract from sorghum bicolor grains. J. Basic Clin. Physiol. Pharmacol..

[21] Wang Z.Y., Huang M.T., Lou Y.R., Xie J.G., Reuhl K.R., Newmark H.L., Ho C.T., Yang C.S., Conney A.H. (1994). Inhibitory effects of black tea, green tea, decaffeinated black tea, and decaffeinated green tea on ultraviolet b light-induced skin carcinogenesis in 7,12-dimethylbenz[*a*]anthracene-initiated skh-1 mice. Cancer Res..

[22] Wang Z.Y., Hong J.Y., Huang M.T., Reuhl K.R., Conney A.H., Yang C.S. (1992). Inhibition of n-nitrosodiethylamine- and 4-(methylnitrosamino)-1-(3-pyridyl)-1-butanone-induced tumorigenesis in a/j mice by green tea and black tea. Cancer Res..

[23] Finch C.E., Crimmins E.M. (2004). Inflammatory exposure and historical changes in human life-spans. Science.

[24] Ahmad A., Banerjee S., Wang Z., Kong D., Majumdar A.P., Sarkar F.H. (2009). Aging and inflammation: Etiological culprits of cancer. Curr. Aging Sci..

[25] He Y., Yue Y., Zheng X., Zhang K., Chen S., Du Z. (2015). Curcumin, inflammation, and chronic diseases: How are they linked?. Molecules.

[26] Ercolini A.M., Miller S.D. (2009). The role of infections in autoimmune disease. Clin. Exp. Immunol..

[27] Anand P., Kunnumakkara A.B., Sundaram C., Harikumar K.B., Tharakan S.T., Lai O.S., Sung B., Aggarwal B.B. (2008). Cancer is a preventable disease that requires major lifestyle changes. Pharm. Res..

[28] Tili E., Michaille J.J. (2016). Promiscuous effects of some phenolic natural products on inflammation at least in part arise from their ability to modulate the expression of global regulators, namely micrornas. Molecules.

[29] Schaffer M., Schaffer P.M., Bar-Sela G. (2015). An update on curcuma as a functional food in the control of cancer and inflammation. Curr. Opin. Clin. Nutr. Metab. Care.

[30] Das L., Vinayak M. (2015). Long term effect of curcumin in restoration of tumour suppressor p53 and phase-II antioxidant enzymes via activation of Nrf2 signalling and modulation of inflammation in prevention of cancer. PLoS ONE.

[31] Rana M., Maurya P., Reddy S.S., Singh V., Ahmad H., Dwivedi A.K., Dikshit M., Barthwal M.K. (2016). A standardized chemically modified curcuma longa extract modulates irak-mapk signaling in inflammation and potentiates cytotoxicity. Front Pharmacol..

[32] Ahmad A., Farhan Asad S., Singh S., Hadi S.M. (2000). DNA breakage by resveratrol and cu(II): Reaction mechanism and bacteriophage inactivation. Cancer Lett..

[33] Ahmad A., Syed F.A., Singh S., Hadi S.M. (2005). Prooxidant activity of resveratrol in the presence of copper ions: Mutagenicity in plasmid DNA. Toxicol. Lett..

[34] Inoue H., Nakata R. (2015). Resveratrol targets in inflammation. Endocr. Metab. Immune Disord. Drug Targets.

[35] Latruffe N., Lancon A., Frazzi R., Aires V., Delmas D., Michaille J.J., Djouadi F., Bastin J., Cherkaoui-Malki M. (2015). Exploring new ways of regulation by resveratrol involving mirnas, with emphasis on inflammation. Ann. N. Y. Acad. Sci..

[36] Limagne E., Lancon A., Delmas D., Cherkaoui-Malki M., Latruffe N. (2016). Resveratrol interferes with il1-beta-induced pro-inflammatory paracrine interaction between primary chondrocytes and macrophages. Nutrients.

[37] Shanmugam T., Selvaraj M., Poomalai S. (2016). Epigallocatechin gallate potentially abrogates fluoride induced lung oxidative stress, inflammation via Nrf2/keap1 signaling pathway in rats: An in vivo and in-silico study. Int. Immunopharmacol..

[38] Kweon M.H., Adhami V.M., Lee J.S., Mukhtar H. (2006). Constitutive overexpression of nrf2-dependent heme oxygenase-1 in a549 cells contributes to resistance to apoptosis induced by epigallocatechin 3-gallate. J. Biol. Chem..

[39] Gao D.Q., Qian S., Ju T. (2016). Anticancer activity of honokiol against lymphoid malignant cells via activation of ros-jnk and attenuation of nrf2 and nf-kappab. J. BUON.

[40] Pan S.T., Qin Y., Zhou Z.W., He Z.X., Zhang X., Yang T., Yang Y.X., Wang D., Zhou S.F., Qiu J.X. (2015). Plumbagin suppresses epithelial to mesenchymal transition and stemness via inhibiting Nrf2-mediated signaling pathway in human tongue squamous cell carcinoma cells. Drug Des. Dev. Ther..

[41] Singh B., Shoulson R., Chatterjee A., Ronghe A., Bhat N.K., Dim D.C., Bhat H.K. (2014). Resveratrol inhibits estrogen-induced breast carcinogenesis through induction of nrf2-mediated protective pathways. Carcinogenesis.

[42] Kim J.H., Gupta S.C., Park B., Yadav V.R., Aggarwal B.B. (2012). Turmeric (*Curcuma longa*) inhibits inflammatory nuclear factor (NF)-kappab and nf-kappab-regulated gene products and induces death receptors leading to suppressed proliferation, induced chemosensitization, and suppressed osteoclastogenesis. Mol. Nutr. Food Res..

[43] Pan H., Chen J., Shen K., Wang X., Wang P., Fu G., Meng H., Wang Y., Jin B. (2015). Mitochondrial modulation by epigallocatechin 3-gallate ameliorates cisplatin induced renal injury through decreasing oxidative/nitrative stress, inflammation and NF-kB in mice. PLoS ONE.

[44] Syed D.N., Afaq F., Kweon M.H., Hadi N., Bhatia N., Spiegelman V.S., Mukhtar H. (2007). Green tea polyphenol egcg suppresses cigarette smoke condensate-induced nf-kappab activation in normal human bronchial epithelial cells. Oncogene.

[45] Arora S., Singh S., Piazza G.A., Contreras C.M., Panyam J., Singh A.P. (2012). Honokiol: A novel natural agent for cancer prevention and therapy. Curr. Mol. Med..

[46] Singh T., Katiyar S.K. (2013). Honokiol inhibits non-small cell lung cancer cell migration by targeting pge(2)-mediated activation of beta-catenin signaling. PLoS ONE.

[47] Ahmad A., Banerjee S., Wang Z., Kong D., Sarkar F.H. (2008). Plumbagin-induced apoptosis of human breast cancer cells is mediated by inactivation of nf-kappab and bcl-2. J. Cell Biochem..

[48] Adhami V.M., Afaq F., Ahmad N. (2003). Suppression of ultraviolet b exposure-mediated activation of nf-kappab in normal human keratinocytes by resveratrol. Neoplasia.

[49] Ben-Neriah Y., Karin M. (2011). Inflammation meets cancer, with nf-kappab as the matchmaker. Nat. Immunol..

[50] Sung B., Prasad S., Yadav V.R., Aggarwal B.B. (2012). Cancer cell signaling pathways targeted by spice-derived nutraceuticals. Nutr. Cancer.

[51] Ahmad A., Ginnebaugh K.R., Li Y., Padhye S.B., Sarkar F.H. (2015). Molecular targets of naturopathy in cancer research: Bridge to modern medicine. Nutrients.

[52] Ahmad A., Biersack B., Li Y., Kong D., Bao B., Schobert R., Padhye S.B., Sarkar F.H. (2013). Targeted regulation of pi3k/akt/mtor/nf-kappab signaling by indole compounds and their derivatives: Mechanistic details and biological implications for cancer therapy. Anticancer Agents Med. Chem..

[53] Murtaza I., Adhami V.M., Hafeez B.B., Saleem M., Mukhtar H. (2009). Fisetin, a natural flavonoid, targets chemoresistant human pancreatic cancer aspc-1 cells through dr3-mediated inhibition of NF-kappaB. Int. J. Cancer.

[54] Ahmed S.M., Luo L., Namani A., Wang X.J., Tang X. (2017). Nrf2 signaling pathway: Pivotal roles in inflammation. Biochim. Biophys. Acta.

[55] Qin S., Hou D.X. (2016). Multiple regulations of keap1/Nrf2 system by dietary phytochemicals. Mol. Nutr. Food Res..

[56] Hadi S.M., Ullah M.F., Azmi A.S., Ahmad A., Shamim U., Zubair H., Khan H.Y. (2010). Resveratrol mobilizes endogenous copper in human peripheral lymphocytes leading to oxidative DNA breakage: A putative mechanism for chemoprevention of cancer. Pharm. Res..

[57] Khan H.Y., Zubair H., Ullah M.F., Ahmad A., Hadi S.M. (2011). Oral administration of copper to rats leads to increased lymphocyte cellular DNA degradation by dietary polyphenols: Implications for a cancer preventive mechanism. Biometals.

[58] Zubair H., Azim S., Khan H.Y., Ullah M.F., Wu D., Singh A.P., Hadi S.M., Ahmad A. (2016). Mobilization of intracellular copper by gossypol and apogossypolone leads to reactive oxygen species-mediated cell death: Putative anticancer mechanism. Int. J. Mol. Sci..

[59] Farhan M., Khan H.Y., Oves M., Al-Harrasi A., Rehmani N., Arif H., Hadi S.M., Ahmad A. (2016). Cancer therapy by catechins involves redox cycling of copper ions and generation of reactive oxygen species. Toxins (Basel).

[60] Gupte A., Mumper R.J. (2009). Elevated copper and oxidative stress in cancer cells as a target for cancer treatment. Cancer Treat. Rev..

[61] Yoshino M., Haneda M., Naruse M., Htay H.H., Tsubouchi R., Qiao S.L., Li W.H., Murakami K., Yokochi T. (2004). Prooxidant activity of curcumin: Copper-dependent formation of 8-hydroxy-2′-deoxyguanosine in DNA and induction of apoptotic cell death. Toxicol. In Vitro.

[62] Khan M.A., Gahlot S., Majumdar S. (2012). Oxidative stress induced by curcumin promotes the death of cutaneous t-cell lymphoma (hut-78) by disrupting the function of several molecular targets. Mol. Cancer Ther..

[63] Nazeem S., Azmi A.S., Hanif S., Ahmad A., Mohammad R.M., Hadi S.M., Kumar K.S. (2009). Plumbagin induces cell death through a copper-redox cycle mechanism in human cancer cells. Mutagenesis.

[64] Foundation T.B.N. (1995). Iron as a pro-oxidant. Iron: Nutritional and Physiological Significance the Report of the British Nutrition Foundation’s Task Force.

[65] Ninsontia C., Phiboonchaiyanan P.P., Chanvorachote P. (2016). Zinc induces epithelial to mesenchymal transition in human lung cancer h460 cells via superoxide anion-dependent mechanism. Cancer Cell Int..

[66] Lim J.Y., Kim D., Kim B.R., Jun J.S., Yeom J.S., Park J.S., Seo J.H., Park C.H., Woo H.O., Youn H.S. (2016). Vitamin C induces apoptosis in ags cells via production of ros of mitochondria. Oncol. Lett..

[67] Amatore C., Arbault S., Ferreira D.C.M., Tapsoba I., Verchier Y. (2008). Vitamin c stimulates or attenuates reactive oxygen and nitrogen species (ros, rns) production depending on cell state: Quantitative amperometric measurements of oxidative bursts at plb-985 and raw 264.7 cells at the single cell level. J. Electroanal. Chem..

[68] Du J., Cullen J.J., Buettner G.R. (2012). Ascorbic acid: Chemistry, biology and the treatment of cancer. Biochim. Biophys. Acta.

[69] Bergstrom T., Ersson C., Bergman J., Moller L. (2012). Vitamins at physiological levels cause oxidation to the DNA nucleoside deoxyguanosine and to DNA—Alone or in synergism with metals. Mutagenesis.

[70] Weinberg S.E., Chandel N.S. (2015). Targeting mitochondria metabolism for cancer therapy. Nat. Chem. Biol..

[71] Weber G.F. (2016). Time and circumstances: Cancer cell metabolism at various stages of disease progression. Front. Oncol..

[72] DeBerardinis R.J. (2008). Is cancer a disease of abnormal cellular metabolism? New angles on an old idea. Genet. Med..

[73] Hanhineva K., Torronen R., Bondia-Pons I., Pekkinen J., Kolehmainen M., Mykkanen H., Poutanen K. (2010). Impact of dietary polyphenols on carbohydrate metabolism. Int. J. Mol. Sci..

[74] Qiu P., Sun J., Man S., Yang H., Ma L., Yu P., Gao W. (2017). Curcumin attenuates n-nitrosodiethylamine-induced liver injury in mice by utilizing the method of metabonomics. J. Agric. Food Chem..

[75] Zhang W., Liu Y., Chen X., Bergmeier S.C. (2010). Novel inhibitors of basal glucose transport as potential anticancer agents. Bioorg. Med. Chem. Lett..

[76] Bayet-Robert M., Morvan D. (2013). Metabolomics reveals metabolic targets and biphasic responses in breast cancer cells treated by curcumin alone and in association with docetaxel. PLoS ONE.

[77] Gunnink L.K., Alabi O.D., Kuiper B.D., Gunnink S.M., Schuiteman S.J., Strohbehn L.E., Hamilton K.E., Wrobel K.E., Louters L.L. (2016). Curcumin directly inhibits the transport activity of glut1. Biochimie.

[78] Liao H., Wang Z., Deng Z., Ren H., Li X. (2015). Curcumin inhibits lung cancer invasion and metastasis by attenuating glut1/mt1-mmp/mmp2 pathway. Int. J. Clin. Exp. Med..

[79] Gwak H., Haegeman G., Tsang B.K., Song Y.S. (2015). Cancer-specific interruption of glucose metabolism by resveratrol is mediated through inhibition of akt/glut1 axis in ovarian cancer cells. Mol. Carcinog..

[80] Sinha S., Pal K., Elkhanany A., Dutta S., Cao Y., Mondal G., Iyer S., Somasundaram V., Couch F.J., Shridhar V. (2013). Plumbagin inhibits tumorigenesis and angiogenesis of ovarian cancer cells in vivo. Int. J. Cancer.

[81] Venturelli L., Nappini S., Bulfoni M., Gianfranceschi G., Dal Zilio S., Coceano G., Del Ben F., Turetta M., Scoles G., Vaccari L. (2016). Glucose is a key driver for glut1-mediated nanoparticles internalization in breast cancer cells. Sci. Rep..

[82] Jung K.H., Lee J.H., Park J.W., Moon S.H., Cho Y.S., Choe Y.S., Lee K.H. (2016). Effects of curcumin on cancer cell mitochondrial function and potential monitoring with (1)(8)f-fdg uptake. Oncol. Rep..

[83] Malhotra A., Nair P., Dhawan D.K. (2014). Study to evaluate molecular mechanics behind synergistic chemo-preventive effects of curcumin and resveratrol during lung carcinogenesis. PLoS ONE.

[84] Li W., Ma X., Li N., Liu H., Dong Q., Zhang J., Yang C., Liu Y., Liang Q., Zhang S. (2016). Resveratrol inhibits hexokinases ii mediated glycolysis in non-small cell lung cancer via targeting akt signaling pathway. Exp. Cell Res..

[85] Moreira L., Araujo I., Costa T., Correia-Branco A., Faria A., Martel F., Keating E. (2013). Quercetin and epigallocatechin gallate inhibit glucose uptake and metabolism by breast cancer cells by an estrogen receptor-independent mechanism. Exp. Cell Res..

[86] Wu A.H., Spicer D., Stanczyk F.Z., Tseng C.C., Yang C.S., Pike M.C. (2012). Effect of 2-month controlled green tea intervention on lipoprotein cholesterol, glucose, and hormone levels in healthy postmenopausal women. Cancer Prev. Res. (Phila).

[87] Parimala R., Sachdanandam P. (1993). Effect of plumbagin on some glucose metabolising enzymes studied in rats in experimental hepatoma. Mol. Cell Biochem..

[88] Wu J.P., Zhang W., Wu F., Zhao Y., Cheng L.F., Xie J.J., Yao H.P. (2010). Honokiol: An effective inhibitor of high-glucose-induced upregulation of inflammatory cytokine production in human renal mesangial cells. Inflamm. Res..

[89] Freeman M.R., Kim J., Lisanti M.P., Di Vizio D. (2011). A metabolic perturbation by u0126 identifies a role for glutamine in resveratrol-induced cell death. Cancer Biol. Ther..

[90] Huang Y.T., Lin Y.W., Chiu H.M., Chiang B.H. (2016). Curcumin induces apoptosis of colorectal cancer stem cells by coupling with cd44 marker. J. Agric. Food Chem..

[91] Vander Heiden M.G., Cantley L.C., Thompson C.B. (2009). Understanding the warburg effect: The metabolic requirements of cell proliferation. Science.

[92] Vaughan R.A., Garcia-Smith R., Dorsey J., Griffith J.K., Bisoffi M., Trujillo K.A. (2013). Tumor necrosis factor alpha induces warburg-like metabolism and is reversed by anti-inflammatory curcumin in breast epithelial cells. Int. J. Cancer.

[93] Blanquer-Rossello M.D., Hernandez-Lopez R., Roca P., Oliver J., Valle A. (2017). Resveratrol induces mitochondrial respiration and apoptosis in sw620 colon cancer cells. Biochim. Biophys. Acta.

[94] Potter M., Newport E., Morten K.J. (2016). The warburg effect: 80 years on. Biochem. Soc. Trans..

[95] Lee N., Kim D. (2016). Cancer metabolism: Fueling more than just growth. Mol. Cells.

[96] Chang C.H., Qiu J., O'Sullivan D., Buck M.D., Noguchi T., Curtis J.D., Chen Q., Gindin M., Gubin M.M., van der Windt G.J. (2015). Metabolic competition in the tumor microenvironment is a driver of cancer progression. Cell.

[97] Pistollato F., Giampieri F., Battino M. (2015). The use of plant-derived bioactive compounds to target cancer stem cells and modulate tumor microenvironment. Food Chem. Toxicol..

[98] Sharma S.H., Thulasingam S., Nagarajan S. (2016). Chemopreventive agents targeting tumor microenvironment. Life Sci..

[99] Onishi H., Kai M., Odate S., Iwasaki H., Morifuji Y., Ogino T., Morisaki T., Nakashima Y., Katano M. (2011). Hypoxia activates the hedgehog signaling pathway in a ligand-independent manner by upregulation of smo transcription in pancreatic cancer. Cancer Sci..

[100] Spivak-Kroizman T.R., Hostetter G., Posner R., Aziz M., Hu C., Demeure M.J., Von Hoff D., Hingorani S.R., Palculict T.B., Izzo J. (2013). Hypoxia triggers hedgehog-mediated tumor-stromal interactions in pancreatic cancer. Cancer Res..

[101] Cao L., Xiao X., Lei J., Duan W., Ma Q., Li W. (2016). Curcumin inhibits hypoxia-induced epithelialmesenchymal transition in pancreatic cancer cells via suppression of the hedgehog signaling pathway. Oncol. Rep..

[102] Li W., Cao L., Chen X., Lei J., Ma Q. (2016). Resveratrol inhibits hypoxia-driven ros-induced invasive and migratory ability of pancreatic cancer cells via suppression of the hedgehog signaling pathway. Oncol. Rep..

[103] Averett C., Bhardwaj A., Arora S., Srivastava S.K., Khan M.A., Ahmad A., Singh S., Carter J.E., Khushman M., Singh A.P. (2016). Honokiol suppresses pancreatic tumor growth, metastasis and desmoplasia by interfering with tumor-stromal cross-talk. Carcinogenesis.

[104] Deng P.B., Hu C.P., Xiong Z., Yang H.P., Li Y.Y. (2013). Treatment with egcg in nsclc leads to decreasing interstitial fluid pressure and hypoxia to improve chemotherapy efficacy through rebalance of ang-1 and ang-2. Chin. J. Nat. Med..

[105] Bao B., Ahmad A., Li Y., Azmi A.S., Ali S., Banerjee S., Kong D., Sarkar F.H. (2012). Targeting cscs within the tumor microenvironment for cancer therapy: A potential role of mesenchymal stem cells. Exp. Opin. Ther. Targets.

[106] Buhrmann C., Kraehe P., Lueders C., Shayan P., Goel A., Shakibaei M. (2014). Curcumin suppresses crosstalk between colon cancer stem cells and stromal fibroblasts in the tumor microenvironment: Potential role of emt. PLoS ONE.

[107] Shakibaei M., Kraehe P., Popper B., Shayan P., Goel A., Buhrmann C. (2015). Curcumin potentiates antitumor activity of 5-fluorouracil in a 3d alginate tumor microenvironment of colorectal cancer. BMC Cancer.

[108] Buhrmann C., Shayan P., Kraehe P., Popper B., Goel A., Shakibaei M. (2015). Resveratrol induces chemosensitization to 5-fluorouracil through up-regulation of intercellular junctions, epithelial-to-mesenchymal transition and apoptosis in colorectal cancer. Biochem. Pharmacol..

[109] Lu Y., Miao L., Wang Y., Xu Z., Zhao Y., Shen Y., Xiang G., Huang L. (2016). Curcumin micelles remodel tumor microenvironment and enhance vaccine activity in an advanced melanoma model. Mol. Ther..

[110] Chen L., Yang S., Liao W., Xiong Y. (2015). Modification of antitumor immunity and tumor microenvironment by resveratrol in mouse renal tumor model. Cell Biochem. Biophys..

[111] Lim S.O., Li C.W., Xia W., Cha J.H., Chan L.C., Wu Y., Chang S.S., Lin W.C., Hsu J.M., Hsu Y.H. (2016). Deubiquitination and stabilization of pd-l1 by csn5. Cancer Cell.

[112] Yan W., Wang T.Y., Fan Q.M., Du L., Xu J.K., Zhai Z.J., Li H.W., Tang T.T. (2014). Plumbagin attenuates cancer cell growth and osteoclast formation in the bone microenvironment of mice. Acta Pharmacol. Sin..

[113] Li Z., Xiao J., Wu X., Li W., Yang Z., Xie J., Xu L., Cai X., Lin Z., Guo W. (2012). Plumbagin inhibits breast tumor bone metastasis and osteolysis by modulating the tumor-bone microenvironment. Curr. Mol. Med..

[114] Jang J.Y., Lee J.K., Jeon Y.K., Kim C.W. (2013). Exosome derived from epigallocatechin gallate treated breast cancer cells suppresses tumor growth by inhibiting tumor-associated macrophage infiltration and m2 polarization. BMC Cancer.

[115] Salado C., Olaso E., Gallot N., Valcarcel M., Egilegor E., Mendoza L., Vidal-Vanaclocha F. (2011). Resveratrol prevents inflammation-dependent hepatic melanoma metastasis by inhibiting the secretion and effects of interleukin-18. J. Transl. Med..

[116] Uttarkar S., Piontek T., Dukare S., Schomburg C., Schlenke P., Berdel W.E., Muller-Tidow C., Schmidt T.J., Klempnauer K.H. (2016). Small-molecule disruption of the myb/p300 cooperation targets acute myeloid leukemia cells. Mol. Cancer Ther..

[117] Azim S., Zubair H., Srivastava S.K., Bhardwaj A., Zubair A., Ahmad A., Singh S., Khushman M., Singh A.P. (2016). Deep sequencing and in silico analyses identify myb-regulated gene networks and signaling pathways in pancreatic cancer. Sci. Rep..

[118] Gray A.L., Stephens C.A., Bigelow R.L., Coleman D.T., Cardelli J.A. (2014). The polyphenols (−)-epigallocatechin-3-gallate and luteolin synergistically inhibit tgf-beta-induced myofibroblast phenotypes through rhoa and erk inhibition. PLoS ONE.

[119] Stylos E., Chatziathanasiadou M.V., Syriopoulou A., Tzakos A.G. (2016). Liquid chromatography coupled with tandem mass spectrometry (LC-MS/MS) based bioavailability determination of the major classes of phytochemicals. J. Chromatogr. B Anal. Technol. Biomed. Life Sci..

[120] Maru G.B., Hudlikar R.R., Kumar G., Gandhi K., Mahimkar M.B. (2016). Understanding the molecular mechanisms of cancer prevention by dietary phytochemicals: From experimental models to clinical trials. World J. Biol. Chem..

[121] Faggian M., Sut S., Perissutti B., Baldan V., Grabnar I., Dall'Acqua S. (2016). Natural deep eutectic solvents (nades) as a tool for bioavailability improvement: Pharmacokinetics of rutin dissolved in proline/glycine after oral administration in rats: Possible application in nutraceuticals. Molecules.

[122] Ahmad A., Li Y., Sarkar F.H. (2016). The bounty of nature for changing the cancer landscape. Mol. Nutr. Food Res..

[123] Hackler L., Ozsvari B., Gyuris M., Sipos P., Fabian G., Molnar E., Marton A., Farago N., Mihaly J., Nagy L.I. (2016). The curcumin analog c-150, influencing nf-kappab, upr and akt/notch pathways has potent anticancer activity in vitro and in vivo. PLoS ONE.

[124] Jin R., Xia Y., Chen Q., Li W., Chen D., Ye H., Zhao C., Du X., Shi D., Wu J. (2016). Da0324, an inhibitor of nuclear factor-kappab activation, demonstrates selective antitumor activity on human gastric cancer cells. Drug Des. Dev. Ther..

[125] Liu G.Y., Zhai Q., Chen J.Z., Zhang Z.Q., Yang J. (2016). 2,2'-fluorine mono-carbonyl curcumin induce reactive oxygen species-mediated apoptosis in human lung cancer nci-h460 cells. Eur. J. Pharmacol..

[126] Ye H., Wei X., Wang Z., Zhang S., Ren J., Yao S., Shi L., Yang L., Qiu P., Wu J. (2016). A novel double carbonyl analog of curcumin induces the apoptosis of human lung cancer h460 cells via the activation of the endoplasmic reticulum stress signaling pathway. Oncol. Rep..

[127] Liang B., Liu Z., Cao Y., Zhu C., Zuo Y., Huang L., Wen G., Shang N., Chen Y., Yue X. (2016). Mc37, a new mono-carbonyl curcumin analog, induces g2/m cell cycle arrest and mitochondria-mediated apoptosis in human colorectal cancer cells. Eur. J. Pharmacol..

[128] Hu Y., Zhao C., Zheng H., Lu K., Shi D., Liu Z., Dai X., Zhang Y., Zhang X., Hu W. (2017). A novel stat3 inhibitor ho-3867 induces cell apoptosis by reactive oxygen species-dependent endoplasmic reticulum stress in human pancreatic cancer cells. Anticancer Drugs.

[129] Mohankumar K., Sridharan S., Pajaniradje S., Singh V.K., Ronsard L., Banerjea A.C., Somasundaram D.B., Coumar M.S., Periyasamy L., Rajagopalan R. (2015). Bdmc-a, an analog of curcumin, inhibits markers of invasion, angiogenesis, and metastasis in breast cancer cells via nf-kappab pathway--a comparative study with curcumin. Biomed. Pharmacother..

[130] Sugiyama S., Yoshino Y., Kuriyama S., Inoue M., Komine K., Otsuka K., Kohyama A., Yamakoshi H., Ishioka C., Tanaka M. (2016). A curcumin analog, go-y078, effectively inhibits angiogenesis through actin disorganization. Anticancer Agents Med. Chem..

[131] Faiao-Flores F., Quincoces Suarez J.A., Fruet A.C., Maria-Engler S.S., Pardi P.C., Maria D.A. (2015). Curcumin analog dm-1 in monotherapy or combinatory treatment with dacarbazine as a strategy to inhibit in vivo melanoma progression. PLoS ONE.

[132] Haque A., Rahman M.A., Fuchs J.R., Chen Z.G., Khuri F.R., Shin D.M., Amin A.R. (2015). Flll12 induces apoptosis in lung cancer cells through a p53/p73-independent but death receptor 5-dependent pathway. Cancer Lett..

[133] Shen T., Jiang T., Long M., Chen J., Ren D.M., Wong P.K., Chapman E., Zhou B., Zhang D.D. (2015). A curcumin derivative that inhibits vinyl carbamate-induced lung carcinogenesis via activation of the nrf2 protective response. Antioxid. Redox. Signal.

[134] Gundewar C., Ansari D., Tang L., Wang Y., Liang G., Rosendahl A.H., Saleem M.A., Andersson R. (2015). Antiproliferative effects of curcumin analog l49h37 in pancreatic stellate cells: A comparative study. Ann. Gastroenterol..

[135] Ahmad A., Sayed A., Ginnebaugh K.R., Sharma V., Suri A., Saraph A., Padhye S., Sarkar F.H. (2015). Molecular docking and inhibition of matrix metalloproteinase-2 by novel difluorinatedbenzylidene curcumin analog. Am. J. Transl. Res..

[136] Roy S., Yu Y., Padhye S.B., Sarkar F.H., Majumdar A.P. (2013). Difluorinated-curcumin (cdf) restores pten expression in colon cancer cells by down-regulating mir-21. PLoS ONE.

[137] Bao B., Ali S., Banerjee S., Wang Z., Logna F., Azmi A.S., Kong D., Ahmad A., Li Y., Padhye S. (2012). Curcumin analogue cdf inhibits pancreatic tumor growth by switching on suppressor micrornas and attenuating ezh2 expression. Cancer Res..

[138] Kanwar S.S., Yu Y., Nautiyal J., Patel B.B., Padhye S., Sarkar F.H., Majumdar A.P. (2011). Difluorinated-curcumin (cdf): A novel curcumin analog is a potent inhibitor of colon cancer stem-like cells. Pharm. Res..

[139] Chen M., Zhou B., Zhong P., Rajamanickam V., Dai X., Karvannan K., Zhou H., Zhang X., Liang G. (2016). Increased intracellular reactive oxygen species mediates the anti-cancer effects of wz35 via activating mitochondrial apoptosis pathway in prostate cancer cells. Prostate.

[140] Zou P., Zhang J., Xia Y., Kanchana K., Guo G., Chen W., Huang Y., Wang Z., Yang S., Liang G. (2015). Ros generation mediates the anti-cancer effects of wz35 via activating jnk and er stress apoptotic pathways in gastric cancer. Oncotarget.

[141] Zhang X., Chen M., Zou P., Kanchana K., Weng Q., Chen W., Zhong P., Ji J., Zhou H., He L. (2015). Curcumin analog wz35 induced cell death via ros-dependent er stress and g2/m cell cycle arrest in human prostate cancer cells. BMC Cancer.

[142] Zhao R., Tin L., Zhang Y., Wu Y., Jin Y., Jin X., Zhang F., Li X. (2016). Ef24 suppresses invasion and migration of hepatocellular carcinoma cells in vitro via inhibiting the phosphorylation of src. Biomed. Res. Int..

[143] Zhang P., Bai H., Liu G., Wang H., Chen F., Zhang B., Zeng P., Wu C., Peng C., Huang C. (2015). Microrna-33b, upregulated by ef24, a curcumin analog, suppresses the epithelial-to-mesenchymal transition (emt) and migratory potential of melanoma cells by targeting hmga2. Toxicol. Lett..

[144] He G., Feng C., Vinothkumar R., Chen W., Dai X., Chen X., Ye Q., Qiu C., Zhou H., Wang Y. (2016). Curcumin analog ef24 induces apoptosis via ros-dependent mitochondrial dysfunction in human colorectal cancer cells. Cancer Chemother. Pharmacol..

[145] Yin D.L., Liang Y.J., Zheng T.S., Song R.P., Wang J.B., Sun B.S., Pan S.H., Qu L.D., Liu J.R., Jiang H.C. (2016). Ef24 inhibits tumor growth and metastasis via suppressing nf-kappab dependent pathways in human cholangiocarcinoma. Sci. Rep..

[146] Yar Saglam A.S., Yilmaz A., Onen H.I., Alp E., Kayhan H., Ekmekci A. (2016). Hdac inhibitors, ms-275 and salermide, potentiates the anticancer effect of ef24 in human pancreatic cancer cells. Excli J..

[147] Lu J., Ho C.H., Ghai G., Chen K.Y. (2001). Resveratrol analog, 3,4,5,4′-tetrahydroxystilbene, differentially induces pro-apoptotic p53/bax gene expression and inhibits the growth of transformed cells but not their normal counterparts. Carcinogenesis.

[148] Nam K.A., Kim S., Heo Y.H., Lee S.K. (2001). Resveratrol analog, 3,5,2′,4′-tetramethoxy-trans-stilbene, potentiates the inhibition of cell growth and induces apoptosis in human cancer cells. Arch. Pharm. Res..

[149] Hong Y.B., Kang H.J., Kim H.J., Rosen E.M., Dakshanamurthy S., Rondanin R., Baruchello R., Grisolia G., Daniele S., Bae I. (2009). Inhibition of cell proliferation by a resveratrol analog in human pancreatic and breast cancer cells. Exp. Mol. Med..

[150] Saiko P., Graser G., Giessrigl B., Steinmann M.T., Schuster H., Lackner A., Grusch M., Krupitza G., Jaeger W., Somepalli V. (2013). Digalloylresveratrol, a novel resveratrol analog inhibits the growth of human pancreatic cancer cells. Invest. New Drugs.

[151] Aldawsari F.S., Aguayo-Ortiz R., Kapilashrami K., Yoo J., Luo M., Medina-Franco J.L., Velazquez-Martinez C.A. (2016). Resveratrol-salicylate derivatives as selective dnmt3 inhibitors and anticancer agents. J. Enzyme Inhib. Med. Chem..

[152] Aldawsari F.S., Aguiar R.P., Wiirzler L.A., Aguayo-Ortiz R., Aljuhani N., Cuman R.K., Medina-Franco J.L., Siraki A.G., Velazquez-Martinez C.A. (2016). Anti-inflammatory and antioxidant properties of a novel resveratrol-salicylate hybrid analog. Bioorg. Med. Chem. Lett..

[153] Thomas E., Gopalakrishnan V., Hegde M., Kumar S., Karki S.S., Raghavan S.C., Choudhary B. (2016). A novel resveratrol based tubulin inhibitor induces mitotic arrest and activates apoptosis in cancer cells. Sci. Rep..

[154] Ma Z., Molavi O., Haddadi A., Lai R., Gossage R.A., Lavasanifar A. (2008). Resveratrol analog trans 3,4,5,4′-tetramethoxystilbene (dmu-212) mediates anti-tumor effects via mechanism different from that of resveratrol. Cancer Chemother. Pharmacol..

[155] Cichocki M., Baer-Dubowska W., Wierzchowski M., Murias M., Jodynis-Liebert J. (2014). 3,4,5,4′*-trans*-tetramethoxystilbene (dmu-212) modulates the activation of nf-kappab, ap-1, and stat3 transcription factors in rat liver carcinogenesis induced by initiation-promotion regimen. Mol. Cell Biochem..

[156] Penthala N.R., Thakkar S., Crooks P.A. (2015). Heteroaromatic analogs of the resveratrol analog dmu-212 as potent anti-cancer agents. Bioorg. Med. Chem. Lett..

[157] Jeong N.Y., Yoon Y.G., Rho J.H., Lee J.S., Lee S.Y., Yoo K.S., Song S., Suh H., Choi Y.H., Yoo Y.H. (2011). The novel resveratrol analog hs-1793-induced polyploid lncap prostate cancer cells are vulnerable to downregulation of bcl-xl. Int. J. Oncol..

[158] Jeong M.H., Yang K.M., Choi Y.J., Kim S.D., Yoo Y.H., Seo S.Y., Lee S.H., Ryu S.R., Lee C.M., Suh H. (2012). Resveratrol analog, HS-1793 enhance anti-tumor immunity by reducing the CD4+CD25+ regulatory T cells in FM3A tumor bearing mice. Int. Immunopharmacol..

[159] Jeong S.H., Song I.S., Kim H.K., Lee S.R., Song S., Suh H., Yoon Y.G., Yoo Y.H., Kim N., Rhee B.D. (2012). An analogue of resveratrol hs-1793 exhibits anticancer activity against mcf-7 cells via inhibition of mitochondrial biogenesis gene expression. Mol. Cells.

[160] Jeong S.K., Yang K., Park Y.S., Choi Y.J., Oh S.J., Lee C.W., Lee K.Y., Jeong M.H., Jo W.S. (2014). Interferon gamma induced by resveratrol analog, hs-1793, reverses the properties of tumor associated macrophages. Int. Immunopharmacol..

[161] Kim H.J., Yang K.M., Park Y.S., Choi Y.J., Yun J.H., Son C.H., Suh H.S., Jeong M.H., Jo W.S. (2012). The novel resveratrol analogue hs-1793 induces apoptosis via the mitochondrial pathway in murine breast cancer cells. Int. J. Oncol..

[162] Choi Y.J., Heo K., Park H.S., Yang K.M., Jeong M.H. (2016). The resveratrol analog hs-1793 enhances radiosensitivity of mouse-derived breast cancer cells under hypoxic conditions. Int. J. Oncol..

[163] Waleh N.S., Chao W.R., Bensari A., Zaveri N.T. (2005). Novel d-ring analog of epigallocatechin-3-gallate inhibits tumor growth and vegf expression in breast carcinoma cells. Anticancer Res..

[164] Hashimoto O., Nakamura A., Nakamura T., Iwamoto H., Hiroshi M., Inoue K., Torimura T., Ueno T., Sata M. (2014). Methylated-(3″)-epigallocatechin gallate analog suppresses tumor growth in huh7 hepatoma cells via inhibition of angiogenesis. Nutr. Cancer.

[165] Yu Z., Qin X.L., Gu Y.Y., Chen D., Cui Q.C., Jiang T., Wan S.B., Dou Q.P. (2008). Prodrugs of fluoro-substituted benzoates of egc as tumor cellular proteasome inhibitors and apoptosis inducers. Int. J. Mol. Sci..

[166] Yang H., Sun D.K., Chen D., Cui Q.C., Gu Y.Y., Jiang T., Chen W., Wan S.B., Dou Q.P. (2010). Antitumor activity of novel fluoro-substituted (−)-epigallocatechin-3-gallate analogs. Cancer Lett..

[167] Huo C., Yang H., Cui Q.C., Dou Q.P., Chan T.H. (2010). Proteasome inhibition in human breast cancer cells with high catechol-o-methyltransferase activity by green tea polyphenol egcg analogs. Bioorg. Med. Chem..

[168] Dandawate P., Vemuri K., Venkateswara Swamy K., Khan E.M., Sritharan M., Padhye S. (2014). Synthesis, characterization, molecular docking and anti-tubercular activity of plumbagin-isoniazid analog and its beta-cyclodextrin conjugate. Bioorg. Med. Chem. Lett..

[169] Hahm E.R., Karlsson A.I., Bonner M.Y., Arbiser J.L., Singh S.V. (2014). Honokiol inhibits androgen receptor activity in prostate cancer cells. Prostate.

[170] Bonner M.Y., Karlsson I., Rodolfo M., Arnold R.S., Vergani E., Arbiser J.L. (2016). Honokiol bis-dichloroacetate (honokiol dca) demonstrates activity in vemurafenib-resistant melanoma in vivo. Oncotarget.

[171] Arora S., Tyagi N., Bhardwaj A., Rusu L., Palanki R., Vig K., Singh S.R., Singh A.P., Palanki S., Miller M.E. (2015). Silver nanoparticles protect human keratinocytes against uvb radiation-induced DNA damage and apoptosis: Potential for prevention of skin carcinogenesis. Nanomed.: Nanotechnol. Biol. Med..

[172] Aras A., Khokhar A.R., Qureshi M.Z., Silva M.F., Sobczak-Kupiec A., Pineda E.A., Hechenleitner A.A., Farooqi A.A. (2014). Targeting cancer with nano-bullets: Curcumin, egcg, resveratrol and quercetin on flying carpets. Asian Pac. J. Cancer Prev..

[173] Nayak A.P., Mills T., Norton I. (2016). Lipid based nanosystems for curcumin: Past, present and future. Curr. Pharm. Des..

[174] Rahimi H.R., Nedaeinia R., Sepehri Shamloo A., Nikdoust S., Kazemi Oskuee R. (2016). Novel delivery system for natural products: Nano-curcumin formulations. Avicenna J. Phytomed..

[175] Strojny B., Grodzik M., Sawosz E., Winnicka A., Kurantowicz N., Jaworski S., Kutwin M., Urbanska K., Hotowy A., Wierzbicki M. (2016). Diamond nanoparticles modify curcumin activity: In vitro studies on cancer and normal cells and in ovo studies on chicken embryo model. PLoS ONE.

[176] Yoon I.S., Park J.H., Kang H.J., Choe J.H., Goh M.S., Kim D.D., Cho H.J. (2015). Poly(d,l-lactic acid)-glycerol-based nanoparticles for curcumin delivery. Int. J. Pharm..

[177] Zaman M.S., Chauhan N., Yallapu M.M., Gara R.K., Maher D.M., Kumari S., Sikander M., Khan S., Zafar N., Jaggi M. (2016). Curcumin nanoformulation for cervical cancer treatment. Sci. Rep..

[178] Kheiri Manjili H., Ghasemi P., Malvandi H., Mousavi M.S., Attari E., Danafar H. (2016). Pharmacokinetics and in vivo delivery of curcumin by copolymeric mpeg-pcl micelles. Eur. J. Pharm. Biopharm..

[179] Chen D., Lian S., Sun J., Liu Z., Zhao F., Jiang Y., Gao M., Sun K., Liu W., Fu F. (2016). Design of novel multifunctional targeting nano-carrier drug delivery system based on cd44 receptor and tumor microenvironment ph condition. Drug Deliv..

[180] Ndong Ntoutoume G.M., Granet R., Mbakidi J.P., Bregier F., Leger D.Y., Fidanzi-Dugas C., Lequart V., Joly N., Liagre B., Chaleix V. (2016). Development of curcumin-cyclodextrin/cellulose nanocrystals complexes: New anticancer drug delivery systems. Bioorg. Med. Chem. Lett..

[181] Yallapu M.M., Khan S., Maher D.M., Ebeling M.C., Sundram V., Chauhan N., Ganju A., Balakrishna S., Gupta B.K., Zafar N. (2014). Anti-cancer activity of curcumin loaded nanoparticles in prostate cancer. Biomaterials.

[182] Fatima M.T., Chanchal A., Yavvari P.S., Bhagat S.D., Gujrati M., Mishra R.K., Srivastava A. (2016). Cell permeating nano-complexes of amphiphilic polyelectrolytes enhance solubility, stability, and anti-cancer efficacy of curcumin. Biomacromolecules.

[183] Yan J., Wang Y., Zhang X., Liu S., Tian C., Wang H. (2016). Targeted nanomedicine for prostate cancer therapy: Docetaxel and curcumin co-encapsulated lipid-polymer hybrid nanoparticles for the enhanced anti-tumor activity in vitro and in vivo. Drug Deliv..

[184] Fang X.B., Zhang J.M., Xie X., Liu D., He C.W., Wan J.B., Chen M.W. (2016). Ph-sensitive micelles based on acid-labile pluronic f68-curcumin conjugates for improved tumor intracellular drug delivery. Int. J. Pharm..

[185] Wang J., Ma W., Tu P. (2015). Synergistically improved anti-tumor efficacy by co-delivery doxorubicin and curcumin polymeric micelles. Macromol. Biosci..

[186] Kesharwani P., Banerjee S., Padhye S., Sarkar F.H., Iyer A.K. (2015). Parenterally administrable nano-micelles of 3,4-difluorobenzylidene curcumin for treating pancreatic cancer. Colloids Surf. B Biointerfaces.

[187] Basak S.K., Zinabadi A., Wu A.W., Venkatesan N., Duarte V.M., Kang J.J., Dalgard C.L., Srivastava M., Sarkar F.H., Wang M.B. (2015). Liposome encapsulated curcumin-difluorinated (cdf) inhibits the growth of cisplatin resistant head and neck cancer stem cells. Oncotarget.

[188] Kesharwani P., Banerjee S., Padhye S., Sarkar F.H., Iyer A.K. (2015). Hyaluronic acid engineered nanomicelles loaded with 3,4-difluorobenzylidene curcumin for targeted killing of cd44+ stem-like pancreatic cancer cells. Biomacromolecules.

[189] Kesharwani P., Xie L., Banerjee S., Mao G., Padhye S., Sarkar F.H., Iyer A.K. (2015). Hyaluronic acid-conjugated polyamidoamine dendrimers for targeted delivery of 3,4-difluorobenzylidene curcumin to cd44 overexpressing pancreatic cancer cells. Colloids Surf. B Biointerfaces.

[190] Bisht S., Schlesinger M., Rupp A., Schubert R., Nolting J., Wenzel J., Holdenrieder S., Brossart P., Bendas G., Feldmann G. (2016). A liposomal formulation of the synthetic curcumin analog ef24 (lipo-ef24) inhibits pancreatic cancer progression: Towards future combination therapies. J. Nanobiotechnol..

[191] Zhang J., Li S., An F.F., Liu J., Jin S., Zhang J.C., Wang P.C., Zhang X., Lee C.S., Liang X.J. (2015). Self-carried curcumin nanoparticles for in vitro and in vivo cancer therapy with real-time monitoring of drug release. Nanoscale.

[192] Gupta R.C., Bansal S.S., Aqil F., Jeyabalan J., Cao P., Kausar H., Russell G.K., Munagala R., Ravoori S., Vadhanam M.V. (2012). Controlled-release systemic delivery—A new concept in cancer chemoprevention. Carcinogenesis.

[193] Sanna V., Siddiqui I.A., Sechi M., Mukhtar H. (2013). Resveratrol-loaded nanoparticles based on poly(epsilon-caprolactone) and poly(d,l-lactic-co-glycolic acid)-poly(ethylene glycol) blend for prostate cancer treatment. Mol. Pharm..

[194] Pulliero A., Wu Y., Fenoglio D., Parodi A., Romani M., Soares C.P., Filaci G., Lee J.L., Sinkam P.N., Izzotti A. (2015). Nanoparticles increase the efficacy of cancer chemopreventive agents in cells exposed to cigarette smoke condensate. Carcinogenesis.

[195] Pujara N., Jambhrunkar S., Wong K.Y., McGuckin M., Popat A. (2017). Enhanced colloidal stability, solubility and rapid dissolution of resveratrol by nanocomplexation with soy protein isolate. J. Colloid Interface Sci..

[196] Park S.Y., Chae S.Y., Park J.O., Lee K.J., Park G. (2016). Gold-conjugated resveratrol nanoparticles attenuate the invasion and mmp-9 and cox-2 expression in breast cancer cells. Oncol. Rep..

[197] Han D.W., Matsumura K., Kim B., Hyon S.H. (2008). Time-dependent intracellular trafficking of fitc-conjugated epigallocatechin-3-o-gallate in l-929 cells. Bioorg. Med. Chem..

[198] Shutava T.G., Balkundi S.S., Vangala P., Steffan J.J., Bigelow R.L., Cardelli J.A., O'Neal D.P., Lvov Y.M. (2009). Layer-by-layer-coated gelatin nanoparticles as a vehicle for delivery of natural polyphenols. ACS Nano.

[199] Siddiqui I.A., Mukhtar H. (2010). Nanochemoprevention by bioactive food components: A perspective. Pharm. Res..

[200] Wang D., Taylor E.W., Wang Y., Wan X., Zhang J. (2012). Encapsulated nanoepigallocatechin-3-gallate and elemental selenium nanoparticles as paradigms for nanochemoprevention. Int. J. Nanomed..

[201] Siddiqui I.A., Adhami V.M., Ahmad N., Mukhtar H. (2010). Nanochemoprevention: Sustained release of bioactive food components for cancer prevention. Nutr. Cancer.

[202] Shafiei S.S., Solati-Hashjin M., Samadikuchaksaraei A., Kalantarinejad R., Asadi-Eydivand M., Abu Osman N.A. (2015). Epigallocatechin gallate/layered double hydroxide nanohybrids: Preparation, characterization, and in vitro anti-tumor study. PLoS ONE.

[203] Narayanan S., Mony U., Vijaykumar D.K., Koyakutty M., Paul-Prasanth B., Menon D. (2015). Sequential release of epigallocatechin gallate and paclitaxel from plga-casein core/shell nanoparticles sensitizes drug-resistant breast cancer cells. Nanomed.: Nanotechnol. Biol. Med..

[204] Duraipandy N., Lakra R., Kunnavakkam Vinjimur S., Samanta D., K P.S., Kiran M.S. (2014). Caging of plumbagin on silver nanoparticles imparts selectivity and sensitivity to plumbagin for targeted cancer cell apoptosis. Metallomics.

[205] Appadurai P., Rathinasamy K. (2015). Plumbagin-silver nanoparticle formulations enhance the cellular uptake of plumbagin and its antiproliferative activities. IET Nanobiotechnol..

[206] Nair H.A., Snima K.S., Kamath R.C., Nair S.V., Lakshmanan V.K. (2015). Plumbagin nanoparticles induce dose and ph dependent toxicity on prostate cancer cells. Curr. Drug Deliv..

[207] Gou M., Zheng L., Peng X., Men K., Zheng X., Zeng S., Guo G., Luo F., Zhao X., Chen L. (2009). Poly(epsilon-caprolactone)-poly(ethylene glycol)-poly(epsilon-caprolactone) (pcl-peg-pcl) nanoparticles for honokiol delivery in vitro. Int. J. Pharm..

[208] Wang B., Gou M., Zheng X., Wei X., Gong C., Wang X., Zhao Y., Luo F., Chen L., Qian Z. (2010). Co-delivery honokiol and doxorubicin in mpeg-pla nanoparticles. J. Nanosci. Nanotechnol..

[209] Zheng X., Kan B., Gou M., Fu S., Zhang J., Men K., Chen L., Luo F., Zhao Y., Zhao X. (2010). Preparation of mpeg-pla nanoparticle for honokiol delivery in vitro. Int. J. Pharm..

[210] Qiu N., Cai L.L., Xie D., Wang G., Wu W., Zhang Y., Song H., Yin H., Chen L. (2010). Synthesis, structural and in vitro studies of well-dispersed monomethoxy-poly(ethylene glycol)-honokiol conjugate micelles. Biomed. Mater..

[211] Alaarg A., Jordan N.Y., Verhoef J.J., Metselaar J.M., Storm G., Kok R.J. (2016). Docosahexaenoic acid liposomes for targeting chronic inflammatory diseases and cancer: An in vitro assessment. Int. J. Nanomed..

[212] Molfino A., Amabile M.I., Monti M., Arcieri S., Rossi Fanelli F., Muscaritoli M. (2016). The role of docosahexaenoic acid (dha) in the control of obesity and metabolic derangements in breast cancer. Int. J. Mol. Sci..

[213] Wan X.H., Fu X., Ababaikeli G. (2016). Docosahexaenoic acid induces growth suppression on epithelial ovarian cancer cells more effectively than eicosapentaenoic acid. Nutr. Cancer.

[214] Harvey K.A., Xu Z., Saaddatzadeh M.R., Wang H., Pollok K., Cohen-Gadol A.A., Siddiqui R.A. (2015). Enhanced anticancer properties of lomustine in conjunction with docosahexaenoic acid in glioblastoma cell lines. J. Neurosurg..

[215] Roy J., Oliveira L.T., Oger C., Galano J.M., Bultel-Ponce V., Richard S., Guimaraes A.G., Vilela J.M., Andrade M.S., Durand T. (2015). Polymeric nanocapsules prevent oxidation of core-loaded molecules: Evidence based on the effects of docosahexaenoic acid and neuroprostane on breast cancer cells proliferation. J. Exp. Clin. Cancer Res..

[216] Zou L., Zheng B., Zhang R., Zhang Z., Liu W., Liu C., Xiao H., McClements D.J. (2016). Enhancing the bioaccessibility of hydrophobic bioactive agents using mixed colloidal dispersions: Curcumin-loaded zein nanoparticles plus digestible lipid nanoparticles. Food Res. Int..

[217] Neves A.R., Lucio M., Martins S., Lima J.L., Reis S. (2013). Novel resveratrol nanodelivery systems based on lipid nanoparticles to enhance its oral bioavailability. Int. J. Nanomed..

[218] Niu Y., Bai J., Kamm R.D., Wang Y., Wang C. (2014). Validating antimetastatic effects of natural products in an engineered microfluidic platform mimicking tumor microenvironment. Mol. Pharm..

[219] Siddiqui I.A., Adhami V.M., Bharali D.J., Hafeez B.B., Asim M., Khwaja S.I., Ahmad N., Cui H., Mousa S.A., Mukhtar H. (2009). Introducing nanochemoprevention as a novel approach for cancer control: Proof of principle with green tea polyphenol epigallocatechin-3-gallate. Cancer Res..

[220] Siddiqui I.A., Bharali D.J., Nihal M., Adhami V.M., Khan N., Chamcheu J.C., Khan M.I., Shabana S., Mousa S.A., Mukhtar H. (2014). Excellent anti-proliferative and pro-apoptotic effects of (−)-epigallocatechin-3-gallate encapsulated in chitosan nanoparticles on human melanoma cell growth both in vitro and in vivo. Nanomed.: Nanotechnol. Biol. Med..

[221] Tyagi N., Srivastava S.K., Arora S., Omar Y., Ijaz Z.M., Al-Ghadhban A., Deshmukh S.K., Carter J.E., Singh A.P., Singh S. (2016). Comparative analysis of the relative potential of silver, zinc-oxide and titanium-dioxide nanoparticles against uvb-induced DNA damage for the prevention of skin carcinogenesis. Cancer Lett..

[222] Ahmed S., Annu, Chaudhry S.A., Ikram S. (2017). A review on biogenic synthesis of zno nanoparticles using plant extracts and microbes: A prospect towards green chemistry. J. Photochem. Photobiol. B.

[223] Lam P.L., Wong W.Y., Bian Z., Chui C.H., Gambari R. (2017). Recent advances in green nanoparticulate systems for drug delivery: Efficient delivery and safety concern. Nanomedicine (Lond.).

